# Evading Capture by Residual Disease Monitoring: Extramedullary Manifestation of *JAK2* V617F-Positive Primary Myelofibrosis After Allogeneic Stem Cell Transplantation

**DOI:** 10.1155/2015/703457

**Published:** 2015-08-09

**Authors:** Stephen E. Langabeer, James Nolan, Karl Haslam, Lindsey Clarke, Richard Flavin, Eibhlin Conneally

**Affiliations:** ^1^Cancer Molecular Diagnostics, St. James's Hospital, Dublin 8, Ireland; ^2^Department of Haematology, St. James's Hospital, Dublin 8, Ireland; ^3^Department of Histopathology, St. James's Hospital, Dublin 8, Ireland

## Abstract

Monitoring of the *JAK2* V617F allele burden in myeloproliferative neoplasms after allogeneic stem cell transplantation is useful to determine levels of residual disease and has the potential to detect early relapse and guide subsequent clinical intervention. A case is described of a *JAK2* V617F-positive primary myelofibrosis patient who underwent allogeneic stem cell transplantation. Prospective residual disease monitoring of the peripheral blood failed to detect an extramedullary manifestation of the disease, a periorbital myeloid sarcoma, arising nearly three years after transplant. This case serves to highlight a pitfall in residual disease monitoring for myeloproliferative neoplasm-associated mutations in the post-allogeneic stem cell transplantation setting.

## 1. Introduction

Primary myelofibrosis (PMF) is one of the classical Philadelphia chromosome-negative myeloproliferative neoplasms and is clinically characterized by cytopenias, splenomegaly, and multiple disease-related symptoms that reduce the quality of life. The clinical course of PMF is variable with an increased risk of progression to acute myeloid leukemia (AML) as compared to essential thrombocythemia and polycythemia vera [1]. While the introduction of JAK2 inhibitors has improved the outlook for many patients with PMF [2], the only curative option that remains is allogeneic stem cell transplantation (ASCT). Improvements in candidate patient selection and stratification, timing of transplantation, and conditioning regimens have significantly reduced the transplant related morbidity and increased the overall survival for patients undergoing this procedure [3]. The most commonly observed acquired mutations in PMF are the* JAK2* V617F,* CALR* exon 9 mutations, and* MPL* exon 10 mutations with* JAK2* V617F and* CALR* mutation status indicative of overall survival after ASCT [4, 5]. Tracking disease burden utilising these patient-specific mutations provides an individualized approach allowing assessment of disease clearance and providing a marker with which to guide adoptive immunotherapy [6–9].

A case is described in which* JAK2* V617F residual disease monitoring failed to detect an extramedullary manifestation of PMF in a patient after ASCT and which serves to highlight a pitfall of this approach.

## 2. Case Report

Whilst undergoing imaging of a knee replacement for osteoarthritis three years previously, a 54-year-old male was noted to have patchy skeletal sclerosis. Further investigations revealed splenomegaly with a nine-month history of progressive constitutional symptoms (generalised unwellness), lethargy, night sweats, and weight loss. Initial hematological investigation revealed a hemoglobin of 10.3 g/dL, a white cell count of 7.8 × 10^9^/L, platelets of 53 × 10^9^/L, and elevated reticulocyte count (212 × 10^9^/L) and lactate dehydrogenase (LDH; 990 IU/L). The blood film was leukoerythroblastic with red cell anisopoikilocytosis, tear drop cells, basophilic stippling, and 2% circulating blasts. Bone marrow aspirate was unsuccessful; however the bone marrow biopsy showed abnormal marrow architecture and grade IV reticulin fibrosis. The* JAK2* V617F mutation was detected by qualitative PCR confirming the diagnosis of PMF. Four months later the platelet count had fallen to 24 × 10^9^/L with 3% blast cells in the peripheral blood. The LDH was markedly elevated at 1912 IU/L with the patient now noted to have a splenomegaly 12 cm below the costal margin. His risk status based on International Prognostic Scoring System was intermediate-2 [10].

The patient proceeded to reduced intensity conditioning ASCT [11] with a matched sibling donor. The posttransplant course was complicated by* Klebsiella* bacteremia and grade II skin graft-versus-host disease (GVHD). With the introduction of corticosteroids the patient developed* Cytomegalovirus* reactivation requiring readmission, steroid-induced hypomania, and steroid-induced insulin-dependent diabetes mellitus. A robust and validated quantitative PCR (qPCR) assay for* JAK2* V617F [12–14] was employed to determine the allele burden before ASCT (30.7%). At two months after ASCT donor chimerism (DC) was 97% and* JAK2* V617F 0.46% and at six months after ASCT DC was 96% and* JAK2* V617F 0.79%. Full DC (100%) and undetectable* JAK2* V61F were concurrently noted at eleven months after ASCT and remained so at prospective three monthly assessments.

**Figure 1:**
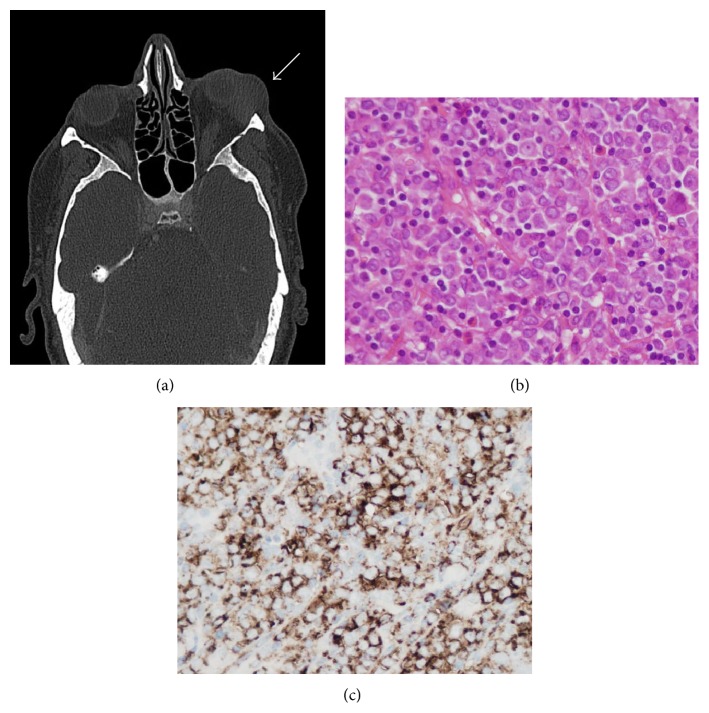
(a) CT showing left periorbital myeloid sarcoma (arrowed); (b) periorbital biopsy showing blast infiltration by myeloid sarcoma (Hematoxylin and Eosin; magnification ×40); (c) positive immunohistochemical staining for CD34 in the myeloid sarcoma (magnification ×40).

In the long-term follow-up clinic at 32 months after ASCT, a 1 cm hard lump was noted beneath the left eye. Subsequent imaging noted a mass lesion arising within the inferolateral aspect of the left orbit, displacing the globe anteromedially and which appeared to lie within the extraconal space (Figure 1(a)). Excision of the periorbital bulk mass was undertaken with histopathological examination revealing adipose/connective tissue infiltrated by blasts (Figure 1(b)) which stained positively for CD34, CD117, and CD45 (Figure 1(c)) and negatively for CD20, TdT, Granzyme B, CD68, and CD1a. The blasts were also* JAK2* V617F-positive consistent with a diagnosis of myeloid sarcoma, an extramedullary tumor (EMT). At this time the patient had a hemoglobin level of 14.0 g/dL, white cell count of 4.2 × 10^9^/L, neutrophils of 2.0 × 10^9^/L, and an unchanged, maintained platelet count of 115 × 10^9^/L. The* JAK2* V617F was undetectable in both the peripheral blood and the bone marrow aspirate, the latter showing no morphological evidence of PMF.

The patient was not a candidate for further intensive chemotherapy and underwent involved field radiotherapy to the left orbit (30 Gy in 15 fractions) and at last follow-up 41 months after ASCT, the* JAK2* V617F remained undetectable in the peripheral blood.

## 3. Discussion

Despite the fact that ASCT remains the only curative option for PMF, the five-year overall survival with reduced intensity conditioning is approximately 50%, with relapse being the major cause of treatment failure which is often associated with an aggressive clinical course, primarily due to clonal evolution [15]. Close molecular monitoring after ASCT is employed to determine the rate and status of disease clearance and to predict impending relapse allowing prompt clinical intervention. In those PMF patients in whom sensitive, residual disease monitoring of the* JAK2* V617F allele burden has been performed, reemergence of this clone in either the peripheral blood or bone marrow precedes loss of DC and clinical relapse [16, 17]. A potential confounding factor of this qPCR approach is the possibility of clonal evolution in which the* JAK2* V617F relinquishes its role as the main driving mutation of this disease. While early detection of extramedullary relapse of AML cases using residual disease monitoring of AML-associated genetic markers has been documented, evidence for a similar benefit in* JAK2* V617F-positive PMF, particularly after ASCT, is scarce [18–20].

EMT arising after ASCT for hematological malignancies appears to be predominantly associated with an initial diagnosis of AML but has been noted in association with other myeloid and lymphoid malignancies and they may occur in the skin and soft tissues, central nervous system, and gastrointestinal tract and at other sites [21]. EMTs are due to the migration of abnormal neoplastic hematopoietic precursor cells into the tissues and therefore formation should be considered a metastatic process [22, 23]. EMTs occurring after ASCT may possess different clinical characteristics compared to bone marrow relapses such as a longer duration of onset and an association with GVHD, both apparent in the case described herein [21]. The mechanism(s) by which extramedullary disease relapse remains molecularly undetected in the peripheral blood remains unknown: one possibility is evasion of PMF stem cells from immunological detection by residing in a shielded niche.

Residual disease monitoring using* JAK2* V617F qPCR in PMF after ASCT is effective in predicting relapse in the majority of those patients in whom the disease is driven by this mutation. However, vigilance is required as extramedullary manifestations of PMF, although rare, may evade early capture by this approach.
